# Impact of pneumonitis from radiotherapy combined with immune checkpoint inhibitors therapy on tumor progression and survival in patients with non-small cell lung cancer

**DOI:** 10.3389/fimmu.2025.1578057

**Published:** 2025-05-09

**Authors:** Ziwei Wang, Kunpeng Xu, Han Sun, Jun Liang, Wei Jiang, Luhua Wang

**Affiliations:** ^1^ Oncology, Graduate School of Bengbu Medical University, Bengbu, Anhui, China; ^2^ Department of Radiation Oncology, National Cancer Center/National Clinical Research Center for Cancer/Cancer Hospital & Shenzhen Hospital, Chinese Academy of Medical Sciences and Peking Union Medical College, Shenzhen, China

**Keywords:** treatment-related pneumonitis, NSCLC, thoracic radiotherapy, immune checkpoint inhibitors therapy, prognosis

## Abstract

**Purpose:**

This study evaluates the impact of thoracic radiotherapy (TRT) combined with immune checkpoint inhibitors (ICIs) treatment-related pneumonitis on tumor progression and prognosis in patients with non-small cell lung cancer (NSCLC).

**Methods:**

Data were collected retrospectively from NSCLC patients treated with TRT and ICIs between January 2019 and August 2023. Treatment-related pneumonitis (TRP) was assessed and graded using the Common Terminology Criteria for Adverse Events (CTCAE) and the Chinese Society of Clinical Oncology Guidelines for Managing Immunotherapy-Related Toxicities. Kaplan-Meier curves and log-rank tests examined associations between pneumonitis with local recurrence-free survival (LRFS), distant metastasis-free survival (DMFS), progression-free survival (PFS), and overall survival (OS). COX regression identified prognostic factors in the pneumonitis group.

**Results:**

Among 86 patients, 58 (67.4%) developed TRP, including 37.2% with grade 2 pneumonitis, and no grade ≥3 cases. 12 patients (14.0%) developed mixed radiation and ICIs pneumonitis. The pneumonitis group had significantly shorter DMFS (12.07 vs not reached, p = 0.028) and PFS (9.53 vs 14.27 months, p = 0.040), shorter LRFS compared to the non-pneumonitis group, but similar OS. High-grade pneumonitis correlated with worse outcomes, especially DMFS (p = 0.031), basically no differences among pneumonitis types. Multivariate COX analysis identified solitary pulmonary nodules or masses as independent negative prognostic factors for PFS, while higher MLD (mean lung dose) independently predicted reduced OS.

**Conclusion:**

Pneumonitis resulting from TRT combined with ICIs was associated with shorter PFS but did not affect OS in NSCLC patients. Mixed pneumonitis did not worsen outcomes. Larger prospective studies are needed to validate these findings.

## 1 Introduction

According to the latest GLOBOCAN2022 data, lung cancer remains the leading malignant tumor globally in both morbidity and mortality (1). Non-small cell lung cancer (NSCLC) is the predominant histological type, accounting for 85% of all lung cancer cases. In recent years, immune checkpoint inhibitors (ICIs) have revolutionized NSCLC treatment, establishing it as the first-line standard for advanced driver-negative cases. Moreover, following the landmark PACIFIC trial, durvalumab as a consolidation treatment following concurrent chemoradiotherapy (CCRT) has become the new standard for unresectable locally advanced NSCLC (2).

The combination of radiotherapy (RT) and ICIs therapy has shown a synergistic effect. However, whether used as consolidation in locally advanced settings, or palliative RT for oligo-metastatic cases, this combination increased the risk of toxicity, particularly pneumonitis, which has emerged as a key challenge in clinical practice (3). In the PACIFIC study, combining RT with durvalumab led to a higher incidence of pneumonitis compared to the placebo group (any grade: 33.9% vs. 24.8%; grade 3-4: 3.4% vs. 2.6%) (2). Similarly, in a large cohort of 1994 patients, the 2-year cumulative incidence of grades ≥2 pneumonitis was significantly higher with CRT plus durvalumab (22.1%) than with CRT alone (13.9%; P ≤ 0.001) (4). Grade 2 or higher pneumonitis during durvalumab consolidation therapy after chemoradiotherapy in stage III NSCLC was revealed to be associated with poorer progression-free survival (PFS) and overall survival (OS) (5).

However, reports on the impact of pneumonitis following radiotherapy or checkpoint inhibitors are inconsistent. Severe acute radiation pneumonitis (SARP), defined as grade ≥3, is a potentially life-threatening side effect of thoracic radiotherapy (TRT), diminishing survival benefits and impacting survivors’ quality of life (6–8). The prognostic significance of CIP is controversial. Suresh et al. (9) reported that CIP correlated with worse survival outcomes in advanced NSCLC, while Haratani et al. (10) indicated that irAEs, including CIP, were associated with better OS and PFS.

There is a distinction in the type of pneumonitis that develops in patients receiving a combination of RT and ICIs therapy in a clinical setting. So in this study, we conducted a retrospective analysis to explore the impact of the occurrence, type, and classification of pneumonitis on tumor progression and survival. Additionally, we assessed clinical and imaging predictors of poor prognosis in this patient population.

## 2 Materials and methods

### 2.1 Patients and treatment

This retrospective study was approved by the Ethics Committee of Cancer Hospital & Shenzhen Hospital, Chinese Academy of Medical Sciences. It included NSCLC patients who underwent TRT in combination with ICIs from January 2019 to August 2023. The inclusion criteria were as follows: (1) a confirmed initial NSCLC pathologically diagnosed; (2) administration of TRT and ICIs before the onset of pneumonitis; (3) availability of clinical, pathological, and dosimetric data. The exclusion criteria included: (1) absence of laboratory and imaging evaluations at the time of diagnosing treatment-related pneumonitis (TRP); (2) loss to follow-up; (3) prior TRT for other conditions; (4) asymptomatic pulmonary fibrosis six months after RT without a prior history of acute pneumonitis. Given the favorable prognosis associated with oligometastatic disease, in addition to locally advanced NSCLC, we included stage IV patients with 1–2 oligometastatic lesions.

### 2.2 Data collection and follow-up

Demographic data, clinical characteristics, details on pneumonitis and tumor treatments, radiotherapy dosimetry parameters, inflammation-related testing and imaging within a week of pneumonitis onset and throughout the treatment process were gathered from the patients’ electronic medical records. All patients underwent chest computed tomography scans before and after treatment every 1–3 months, continuing for up to 6 months prior to the final follow-up. Follow-up visits on disease progression and survival, either in-person or via telephone, were conducted every 3 months starting from the initiation of RT. Survival tracking began on the date RT was initiated, with endpoints defined as the time to tumor progression, last follow-up, or death. The primary endpoints were progression-free survival (PFS) and overall survival (OS), while secondary endpoints included local recurrence-free survival (LRFS) and distant metastasis-free survival (DMFS). The final follow-up was scheduled for May 2024.

Additionally, complete blood count data obtained within a week of pneumonitis onset were used to calculate the systemic immune-inflammation index (SII), neutrophil-to-lymphocyte ratio (NLR), and platelet-to-lymphocyte ratio (PLR) using the following formulas: SII = platelet count × neutrophil count/lymphocyte count, NLR = neutrophil count/lymphocyte count, and PLR = platelet count/lymphocyte count.

### 2.3 Diagnosis and assessment of treatment-related pneumonitis

A multidisciplinary oncology team, comprising at least one radiation oncologist and one imaging expert, established the diagnosis, types (radiation pneumonitis, checkpoint inhibitors pneumonitis, and mixed pneumonitis), grading, and imaging characteristics of pneumonitis. The staging followed our previously defined criteria (11). Radiation pneumonitis (RP) typically occurs within six months after RT and is generally aligned with the dose distribution curve, while checkpoint inhibitors pneumonitis (CIP) that arises following ICIs appears more diffuse on CT scans and exhibits various imaging patterns, although the pneumonitis area rarely crosses lung fissures. Mixed pneumonitis is classified when both RP and CIP features are present, cannot be distinctly differentiated, and other causes such as heart failure, infection, and tumor progression have been excluded (12). The Common Terminology Criteria for Adverse Events (CTCAE, v5.0) was utilized to grade pneumonitis, categorizing symptomatic TRP as grade ≥2. All suspected pneumonitis diagnoses were evaluated by multiple physicians, ensuring a consensus conclusion. Imaging characteristics of TRP were categorized based on the International Multidisciplinary Classification Criteria established by the American Thoracic Society and the European Respiratory Society (ATS/ERS). Following prior research, pneumonitis-related imaging patterns were classified into organizing pneumonitis (OP), ground glass opacities (GGO), acute interstitial pneumonitis (AIP), hypersensitivity pneumonitis (HP), non-specific interstitial pneumonitis (NSIP) (13).

### 2.4 Statistical analysis

The data from this study were analyzed using SPSS 27.0 software. Descriptive analysis was conducted for all variables. Categorical variables were summarized as frequencies and percentages, while continuous variables were presented as medians. Receiver Operating Characteristic (ROC) curve analysis was utilized to determine the optimal critical values for each immunoinflammatory index and target area plan parameter. The χ2 test was used for comparing categorical variables, while continuous variables were analyzed using either the independent samples t-test or the nonparametric Mann-Whitney U test. Survival analysis of pneumonitis-related factors was evaluated using the Log-rank test, along with the Kaplan-Meier method for survival curves plotting survival curves. Cox proportional hazards models were applied for multivariate regression analyses to identify potential prognostic factors in the pneumonitis group. Potential interactions between variables were evaluated using the interactionR package in R (version 4.0.0). A p-value of less than 0.05 was deemed statistically significant.

## 3 Results

### 3.1 Patient characteristics

This study included 86 patients with NSCLC who underwent TRT combined with ICIs. The median age of the cohort was 65 years (range: 40–82). Among them, 52 patients (60.5%) had a smoking history, 16 patients (18.6%) had previously undergone lobectomy, and the predominant histological type was squamous cell carcinoma (48,55.8%). Additionally, 54 patients (62.8%) were diagnosed with stage III disease, and 30.2% were in stage IV. In terms of treatment modalities, 25 (29.1%) received CCRT, and 32 (37.2%) underwent RT concurrent ICIs. The clinical characteristics of all patients are presented in **Table 1**.

**Table 1:** Patient baseline characteristics.

Characteristics	N (%) (N = 86)
Sex	
Female	13 (15.1)
Male	73 (84.9)
Median Age, Years (Range)	65 (40-82)
KPS	
≥90	75 (87.2)
<90	11 (12.8)
History of smoking	
No	34 (39.5)
Yes	52 (60.5)
History of chest operation	
No	70 (81.4)
Yes	16 (18.6)
Histological type	
Adenocarcinoma	31 (36.0)
Squamous cell lung carcinoma	48 (55.8)
NSCLC-NOS	7 (8.1)
T stage	
T1-2	35 (40.7)
T3-4	51 (59.3)
N stage	
N0-1	9 (10.5)
N2-3	77 (89.5)
M stage	
M0	60 (69.8)
M1	26 (30.2)
Initial cancer stage	
I-II	6 (7.0)
III	54 (62.8)
IV	26 (30.2)
Comorbidities	
No	51 (59.3)
Yes	35 (40.7)
Concurrent chemoradiotherapy	
No	61 (70.9)
Yes	25 (29.1)
ICIs Timing	
Concurrent	32 (37.2)
Sequential	54 (62.8)

KPS, karnofsky performance status; ICIs, immune checkpoint inhibitors; NSCLC-NOS, non-small cell lung cancer -not otherwise specified.

### 3.2 Features of treatment-related pneumonitis

58 patients (67.4%) experienced TRP, with a median onset time of 3.58 months (ranging from 0.47 to 11.77 months) from the initiation of the first RT. **Supplementary Table 1** outlines the characteristics of TRP: 40 cases (46.5%) were classified as RP, 6 cases (6.98%) as CIP, and 12 cases (14.0%) as mixed pneumonitis. Of these, 32 cases (37.2%) had grade 2 symptomatic pneumonitis, and the remaining 30.2% had grade 1 pneumonitis. No pneumonitis-related deaths were observed. We subsequently compared the clinical features of patients with pneumonitis to those without, as detailed in **Supplementary Table 2**. The median RT dose for the pneumonitis group was 54 Gy (ranging from 30 to 66 Gy), which was comparable to that of patients without pneumonitis. However, both the MLD (P = 0.026) and V20 (P = 0.037) were associated with the development of TRP, showing significantly higher values in the pneumonitis group compared to the non-pneumonitis group. Concurrent chemotherapy or ICIs were also risk factors for pneumonitis.

Patients with TRP exhibited a variety of CT imaging findings (as illustrated in **Supplementary Table 3**). The five most prevalent CT signs were patch (100%), lung consolidation (87.9%), strip shape (79.3%), ground-glass opacity (62.1%), and honeycomb (42.1%). In cases of TRP, synchronous tumor progression was observed in 12.1% of patients, pleural effusion in 31.0%, and enlarged non-neoplastic lymph nodes in hilar and mediastinal regions of the lungs in 84.5%. The predominant imaging pattern was OP (100%), followed by GGO (63.8%), NSIP (13.8%), lung nodules or mass-like (5.2%), HP (3.4%), and only one case (1.7%) of bronchitis. No cases of pneumonitis were presented as AIP or DAD.

### 3.3 Features of treatment-related pneumonitis

The median follow-up period after the initial RT was 24.1 months (ranging from 9.93 to 66.47 months), with a median OS of 20.47 months (95% CI: 20.85-27.03 months) and PFS of 9.70 months (95% CI: 10.13-14.67 months) for all patients.

**Figure 1:**
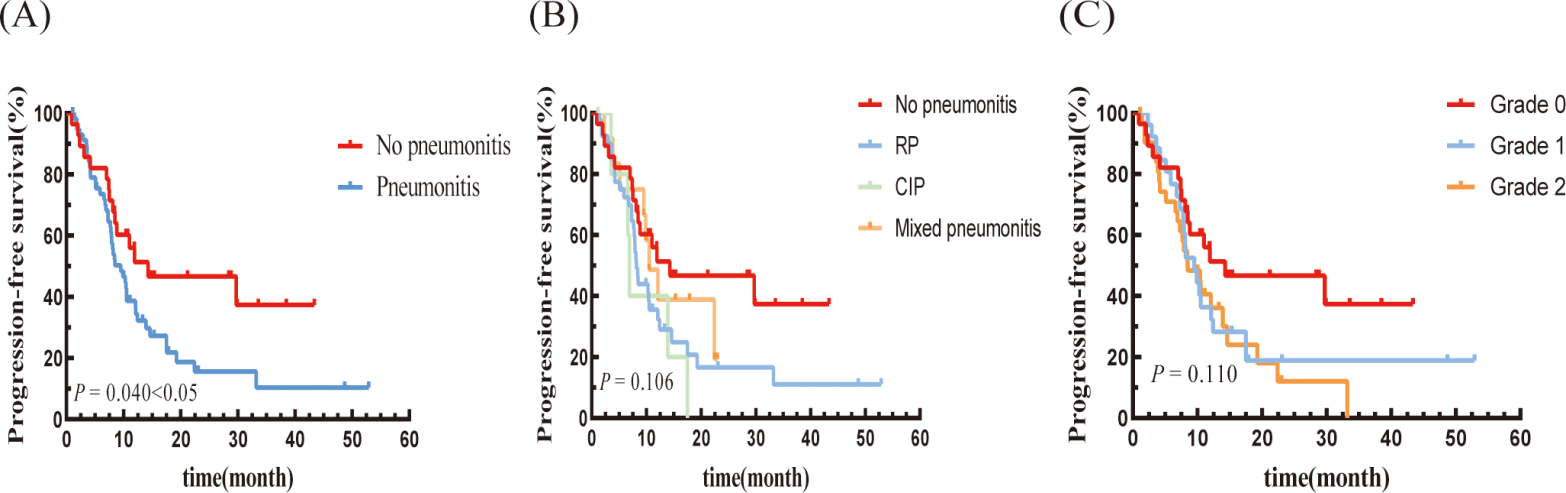
Kaplan–Meier graph of PFS for patients according to **(A)** Pneumonitis or not, **(B)** Type of pneumonitis, and **(C)** Pneumonitis classification.

Patients in the pneumonitis group had a median PFS of 9.53 months (95% CI: 6.90-12.16 months), compared to 14.27 months (95% CI: 0.00-33.96 months) in the non-pneumonitis group (P=0.040; **Figure 1A**). There was no statistically significant difference in PFS among the three types of pneumonitis (**Figure 1B**). Both grade 1 and grade 2 pneumonitis significantly reduced PFS, with no difference between the grades. (**Figure 1C**).

**Figure 2:**
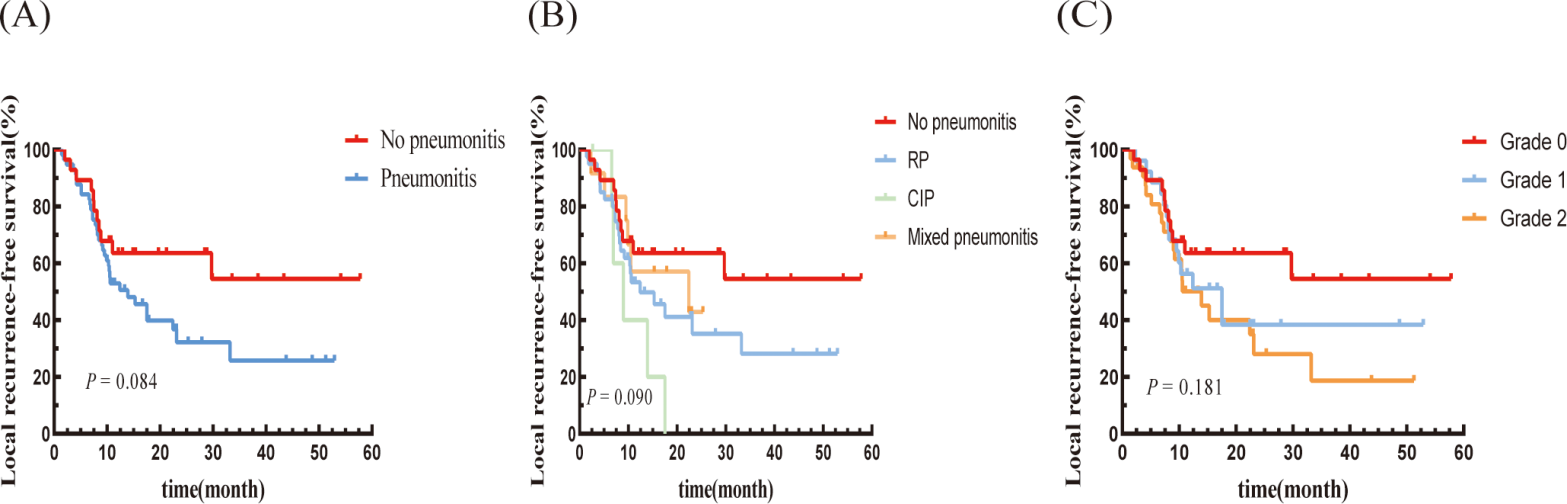
Kaplan–Meier graph of LRFS for patients according to **(A)** Pneumonitis or not, **(B)** Type of pneumonitis, and **(C)** Pneumonitis classification.

The median LRFS for the pneumonitis group was 13.9 months (95% CI: 6.139-21.661 months), which was significantly shorter than that of the non-pneumonitis group (not reached). Although the difference was not statistically significant, it showed a notable downward trend (P=0.084; **Figure 2A**). In terms of pneumonitis type, the CIP group exhibited a lower trend compared to the other two groups. the LRFS trends were consistent with the PFS trends across pneumonitis grades (**Figures 2B, C**).

**Figure 3:**
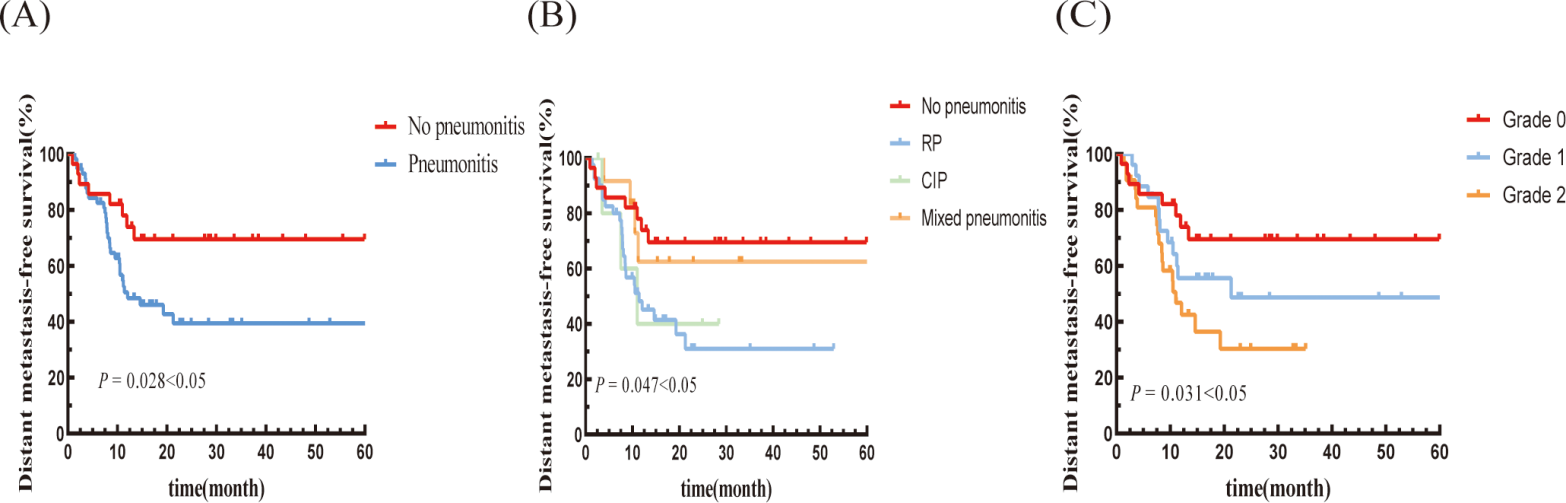
Kaplan–Meier graph of DMFS for patients according to **(A)** Pneumonitis or not, **(B)** Type of pneumonitis, and **(C)** Pneumonitis classification.

The median DMFS was 12.07 months (95% CI: 3.67-20.47 months) in the pneumonitis group, which was shorter than in the non-pneumonitis group (not reached), with a statistically significant difference (P=0.028; **Figure 3A**). The mixed pneumonitis group showed a higher trend in DMFS compared to the other two groups. However, patients with grade 2 pneumonitis had a lower DMFS compared to those with grade 1 pneumonitis and those without pneumonitis, with all differences reaching statistical significance (**Figures 3B, C**).

**Figure 4:**
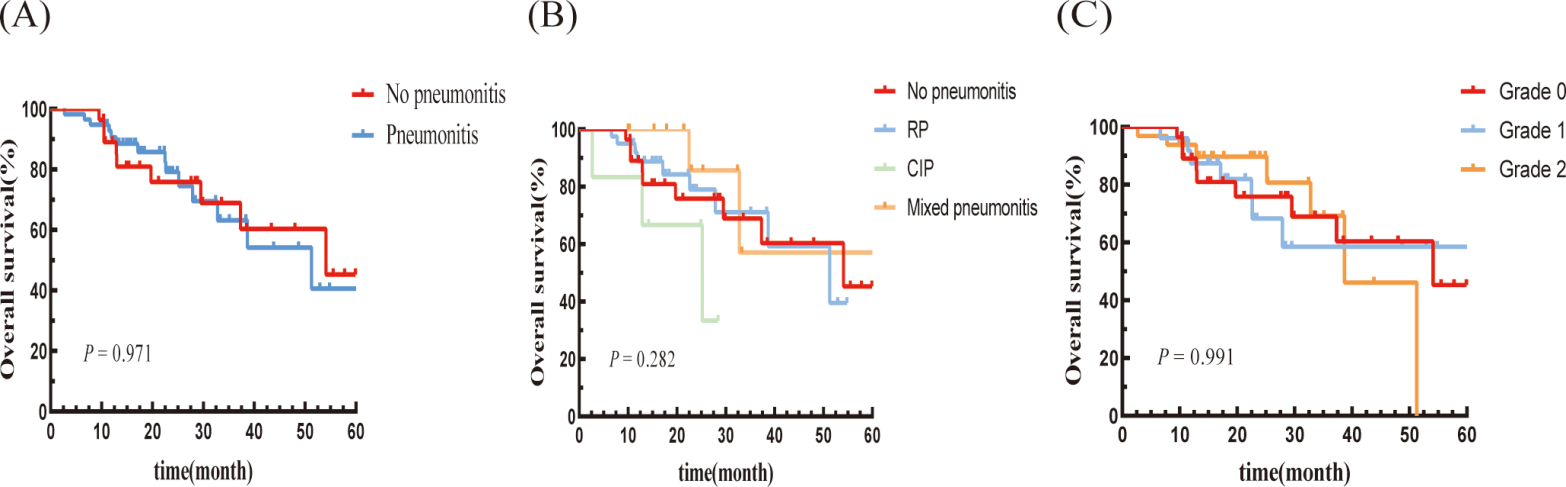
Kaplan–Meier graph of OS for patients according to **(A)** Pneumonitis or not, **(B)** Type of pneumonitis, and **(C)** Pneumonitis classification.

There was no significant difference in OS between the pneumonitis group and the non-pneumonitis group (P = 0.971; **Figure 4A**). Moreover, there were no significant differences in OS based on the type or grade of pneumonitis (**Figures 4B, C**).

### 3.4 Prognostic factors for patients in the pneumonitis group

In 58 patients with pneumonitis, the progression group had a significantly higher NLR within one week (4.41 vs. 2.78, P = 0.02) and a larger PTV for RT (208.6 cm³ vs. 143.1 cm³, P = 0.044) compared to the non-progression group (**Table 2**). No significant associations were observed between tumor progression and clinical features, tumor status, treatment, dosimetric parameters and imaging characteristics (**Supplementary Table 4**).

**Table 2:** Characteristics of patients in the two groups and with the χ2 test for categorical variables.

Variable	Group with tumor progression (n = 44)	Group with no tumor progression (n = 14)	*P* value
WBC,10^9/L (range)	5.53 (2.53-12.72)	4.69 (2.88-8.25)	0.364
NEU,10^9/L (range)	3.45 (1.34-11.07)	2.93 (1.80-5.03)	0.081
LYM,10^9/L (range)	0.84 (0.26-2.39)	0.96 (0.49-2.00)	0.173
MONO,10^9/L (range)	0.58 (0.20-1.59)	0.48 (0.32-0.91)	0.424
PLT,10^9/L (range)	201.00 (53.00-412.00)	219.50 (126.00-335.00)	0.775
NLR	4.41 (0.59-20.97)	2.78 (1.17-6.59)	0.020
PLR	221.97 (96.36-948.80)	206.52 (101.00-544.90)	0.260
SII	918.45 (176.50-5769.52)	505.12 (214.47-1760.02)	0.069
Radiotherapy area			0.459
Primary focus of the lungs	3 (6.8)	1 (7.1)	
Mediastinal lymph nodes	6 (13.6)	0 (0)	
Both	35 (79.5)	13 (92.9)	
PTV, cm3 (range)	208.60 (41.60-467.50)	143.10 (42.80-559.70)	0.044
Median radiotherapy dose,Gy (range)	52.50 (40.00-60.00)	54.00 (45.00-66.00)	0.338
Median Radiotherapy fractionation,T (range)	18 (10-30)	18 (8-30)	0.933
MLD, Gy (range)	983.15 (43.20-1356.90)	946.65 (584.00-1160.80)	0.744
Median V5, %	36.22	33.24	0.962
Median V10, %	26.78	24.62	0.740
Median V20, %	17.52	17.05	0.624
Median V30, %	10.52	10.05	0.758

WBC, White blood count; NEU, neutrophil count; LYM, lymphocyte count; MONO, mononuclear cell count; PLT; Platelet counts; NLR, neutrophil-lymphocyte ratio; PLR, platelet–lymphocyte ratio; SII, systemic immune-inflammation index; PTV, Planning Target Volume; MLD, Mean Lung Dose; V5, percent volume of lung receiving ≥5Gy; V10, percent volume of lung receiving ≥10Gy; V20, percent volume of lung receiving ≥20Gy; V30, percent volume of lung receiving ≥30Gy.

Univariate analysis revealed that NLR ≥ 3.605 (HR: 2.520; 95% CI 1.288-4.931; p = 0.007), SII ≥ 712.46 (HR: 2.058; 95% CI 1.034-4.096; p = 0.04), and the presence of a solitary pulmonary nodule or mass at pneumonitis onset (HR: 2.552, 95% CI: 1.054–6.176, P = 0.038) were factors associated with poor PFS. However, given the existence of interactions, only NLR were included in the multivariate Cox proportional hazards regression based on the interaction results. In the multivariate Cox proportional hazards regression model, a high NLR (HR = 2.841; 95% CI 1.428-5.649; p= 0.003) and the solitary pulmonary nodule or mass (HR: 3.360, 95.0% CI: 1.354-8.340; p=0.009) were identified as independent prognostic factors for PFS (**Table 3**).

**Table 3:** Univariate and multivariate Cox proportional hazards regression for progression free survival in the group with pneumonitis.

Variables	Univariate analysis	*P* value	Multivariate analysis	*P* value
	HR (95% CI)		HR (95% CI)	
Age (≥60 vs. <60)	0.882 (0.459-1.698)	0.708		
Gender (female vs. male)	0.496 (0.230-1.067)	0.073		
Smoking (current or former vs. never)	1.133 (0.620-2.071)	0.684		
KPS (≥90 vs. <90)	2.318 (0.965-5.568)	0.060		
Concurrent chemoradiotherapy (No vs. Yes)	0.967 (0.526-1.778)	0.915		
ICIs therapy types (concurrent vs. sequential)	1.242 (0.679-2.272)	0.481		
Comorbidities (No vs. Yes)	1.360 (0.751-2.465)	0.310		
Pneumonitis leads to interruption of treatment (No vs. Yes)	0.778 (0.370-1.635)	0.508		
NEU (<2.715 vs. ≥2.715)	1.950 (0.864-4.400)	0.108		
NLR (<3.605 vs. ≥3.605)	2.520 (1.288-4.931)	0.007	2.841 (1.428-5.649)	0.003
SII (<712.46 vs. ≥712.46)	2.058 (1.034-4.096)	0.040		
PTV (<191.1 vs. ≥191.1)	1.311 (0.719-2.389)	0.377		
ground-glass opacity (No vs. Yes)	1.764 (0.941-3.308)	0.077		
Thickening of the interlobular septa of the lungs (No vs. Yes)	1.438 (0.785-2.635)	0.239		
Solitary pulmonary nodule or masses (No vs. Yes)	2.552 (1.054-6.176)	0.038	3.360 (1.354-8.340)	0.009
Thickening of lung bronchial walls (No vs. Yes)	1.742 (0.720-4.216)	0.218		
Distribution of pneumonitis (unifocal vs. multifocal)	2.258 (0.877-5.809)	0.091		
GGO (No vs. Yes)	1.852 (0.976-3.512)	0.059		
Lung nodule or mass-like	2.980 (0.906-9.802)	0.072		

KPS, karnofsky performance status; ICIs, immune checkpoint inhibitors; NEU, neutrophil count; NLR, neutrophil-lymphocyte ratio; SII, systemic immune-inflammation index; PTV, Planning Target Volume; GGO, Ground Glass Opacity.

Univariate analysis showed that a MLD ≥ 1091.25 (HR = 4.590; 95% CI 1.427-14.768; p= 0.011), a KPS score < 90 (HR = 3.577; 95% CI 1.092-11.583; p= 0.035), and multifocal pneumonitis distribution (No vs Yes; HR: 6.815, 95.0% CI: 1.679-27.661; p=0.007) were associated with poor OS. In the multivariate analysis, only a high MLD (HR = 4.076; 95% CI 1.186-14.005; p= 0.026) remained an independent prognostic factor for OS in patients with pneumonitis (**Table 4**).

**Table 4:** Univariate and multivariate Cox proportional hazards regression for Overall survival in the group with pneumonitis.

Variables	Univariate analysis	*P* value	Multivariate analysis	*P* value
	HR (95% CI)		HR (95% CI)	
Age (≥60 vs. <60)	0.737 (0.227-2.390)	0.611		
Gender (female vs. male)	0.596 (0.124-2.864)	0.518		
Smoking (current or former vs. never)	0.595 (0.160-2.207)	0.438		
KPS (≥90 vs. <90)	3.577 (1.092-11.583)	0.035	2.377 (0.386-14.647)	0.351
Concurrent chemoradiotherapy (No vs. Yes)	0.823 (0.283-2.392)	0.720		
ICIs therapy types (concurrent vs. sequential)	1.480 (0.472-4.645)	0.502		
Comorbidities (No vs. Yes)	1.772 (0.606-5.179)	0.296		
Pneumonitis leads to interruption of treatment (No vs. Yes)	1.656 (0.518-5.299)	0.395		
V20 (<18.425 vs. ≥18.425)	1.947 (0.638-5.942)	0.242		
V30 (<13.085 vs. ≥13.085)	2.557 (0.870-7.513)	0.088		
MLD (<1091.25 vs. ≥1091.25)	4.590 (1.427-14.768)	0.011	4.076 (1.186-14.005)	0.026
honeycomb (No vs. Yes)	1.394 (0.478-4.063)	0.543		
Synchronized tumor progression (No vs. Yes)	2.085 (0.450-9.649)	0.347		
Thickening of the interlobular septa of the lungs (No vs. Yes)	1.766 (0.615-5.069)	0.291		
Solitary pulmonary nodule or masses (No vs. Yes)	2.498 (0.660-9.463)	0.178		
Thickening of lung bronchial walls (No vs. Yes)	1.833 (0.502-6.683)	0.359		
Distribution of pneumonitis (unifocal vs. multifocal)	6.815 (1.679-27.661)	0.007	2.237 (0.261-19.205)	0.463
NSIP (No vs. Yes)	2.047 (0.637-6.581)	0.229		
Lung nodule or mass-like	3.497 (0.752-16.256)	0.110		

KPS, karnofsky performance status; ICIs, immune checkpoint inhibitors; MLD, Mean Lung Dose; V20, percent volume of lung receiving ≥20Gy; V30, percent volume of lung receiving ≥30Gy; NSIP, Non-specific interstitial pneumonitis.

## 4 Discussion

Our findings, together with that of Kinehara Y et al. (5) both highlighted the adverse impact of RT combined with ICIs therapy-related pneumonitis on PFS. A plausible immunological mechanism underlying this phenomenon involves radiotherapy-induced inflammation triggering systemic immune hyperactivation, leading to excessive cytokine release. This pathological process can severely impair the function of tumor-infiltrating lymphocytes (TILs), result in elevated immunosuppressive factors, and promote a more immunosuppressive microenvironment (14–16). Further mechanistic exploration is needed to validate this hypothesis, particularly regarding immune microenvironment dynamics and cytokine profiling. However, accumulating evidence has consistently demonstrated the detrimental effects of inflammation on tumor progression, underscoring the clinical importance of pneumonitis prevention and active management.

Regarding the impact of pneumonitis on OS, we do not fully align with the conclusions from Kinehara Y et al. (5) While our findings indicate that pneumonitis correlates with poorer progression in NSCLC, its association with OS appears less pronounced. In our study, the absence of grade 3 or higher pneumonitis may account for the absence of survival detriment effects. This observation aligns with previous studies about CIP, where grade 1–2 CIP was reported to be associated with favorable OS, whereas grade 3–4 CIP is not (17), indirectly supporting the above rationale.

Our study indicated a trend towards a relatively poorer prognosis in cases of pure CIP, while mixed pneumonitis did not significantly worsen tumor prognosis. In our research, among the six CIP patients, most (83.3%) had grade 2 symptomatic pneumonitis, whereas RP and mixed pneumonitis were predominantly low-grade and asymptomatic. So this discrepancy may be partly attributed to the frequent treatment interruption or discontinuation in CIP patients, as well as the use of glucocorticoid which has been reported to compromise survival benefits (18).

Follow-up analyses of the PACIFIC study showed that Grade 2+ pneumonitis/radiation pneumonitis (RP) does not compromise the clinical benefits of durvalumab in terms of PFS and OS in patients with unresectable stage III NSCLC (19). This aligns with our findings, indicating that while TRP may increase treatment-related risk and complicated management, it does not significantly affect the long-term OS for patients with NSCLC undergoing RT combined with ICIs. These findings suggested that while proactive management of TRP is essential, the therapeutic potential of this combination strategy should not be disregarded in eligible patient populations.

Our findings revealed that elevated NLR and SII at the onset of pneumonitis were associated with poorer PFS. Both NLR and SII are well-established markers of inflammatory response and immune status. Numerous studies have demonstrated that elevated pretreatment NLR and PLR correlate with shorter PFS and OS in lung cancer patients receiving ICIs (20, 21). Similarly, the predictive value of SII in the PFS of lung cancer patients treated with ICIs was also reported (22). Our previous work using animal models found that the combination of radiotherapy and ICIs exaggerated pulmonary inflammation, with significantly increased levels of neutrophilic infiltration (23).

Furthermore, the study identified that pneumonitis presenting as solitary pulmonary nodule or masses was a predictor of shorter PFS. Solitary pulmonary nodules or masses may be associated with the persistent stimulation of immune responses, but the mechanistic characteristics of TRP with different imaging features remain unclear. Additionally, solitary pulmonary nodules or masses appear to be more commonly observed in CIP compared to RP or mixed-pattern pneumonitis. In this study, the relatively higher severity and poorer prognosis of CIP may explain the correlation between this imaging feature and prognosis. However, due to the small sample size, we may not have identified the true radiological features associated with poor prognosis. Larger sample studies are needed to provide more definitive insights for future clinical practice.

Multifocal imaging patterns have been identified as a prognostic factor for poorer OS in patients with pneumonitis. The multifocal distribution of pneumonitis leads to more extensive and widespread inflammation compared to unifocal patterns, which not only exert multifaceted effects on the tumor immune microenvironment and systemic immunity but also complicates treatment approaches. Furthermore, it increases the risk of complications and secondary health issues, collectively contributing to the observed decline in survival rates.

Consistent with previous reports, CCRT and RT concurrent ICIs therapy in the treatment of NSCLC is more likely to result in TRP (24, 25). However, when dosimetric high-risk factors such as V20 and MLD are controlled, the overall severity of pneumonitis remains mild. According to our findings and previous studies, the impact of mild pneumonitis on tumor progression and survival is acceptable (4, 18). Research indicates that compared to the pre-immunotherapy era, where V20 limits were strictly controlled during CCRT, a V20 exceeding 20% in RT combined with ICIs significantly increases the risk of pneumonitis (26, 27). Therefore, while there is no need for undue hesitation, careful and cautious advancement is still necessary.

However, this study has several limitations that should be acknowledged: the limited number of patients in this study, with *even* six patients in CIP subgroups, affected the ability to confirm statistical significance, which will result in regarding potential type II errors and the reduced generalizability of subgroup analyses. The small number of cases with different types of pneumonitis and varying pneumonitis characteristics precluded drawing definitive clinical implications. Additionally, as a retrospective analysis, this study has inherent limitations. For example, to mitigate the toxicity associated with the combination of RT and ICIs therapy, clinical practice naturally involved cautious strategies to minimize normal lung dose exposure. As a result, the overall severity of pneumonitis observed in this study was relatively mild, with few cases of grade 2 pneumonitis. The true impact of pneumonitis on tumor progression and prognosis, therefore, requires further validation. Furthermore, to increase the sample size, the study included both locally advanced patients and stage IV oligometastatic patients, whose differing disease stages could potentially influence the prognostic data.

## 5 Conclusion

Pneumonitis resulting from the combination of RT and ICIs therapy did not impact OS in NSCLC patients, although it was associated with shorter PFS. Mixed pneumonitis did not further deteriorate the patient’s prognosis. Nevertheless, due to the limited number of cases analyzed thus far, further large-scale prospective studies are necessary to validate these findings and to comprehensively explore risk factors predicting poor prognosis.

## Data Availability

The original contributions presented in the study are included in the article/Supplementary Material. Further inquiries can be directed to the corresponding authors.
