# Supplementary material for: Impact of pneumonitis from radiotherapy combined with immune checkpoint inhibitors therapy on tumor progression and survival in patients with non-small cell lung cancer
Source: Front Immunol. 2025 May 9;16:1578057. doi: 10.3389/fimmu.2025.1578057 (PMC12098286; doi:10.3389/fimmu.2025.1578057)
Supplement: Supplementary file 1 [file Table1.docx]

Supplementary Material

# Supplementary Tables

# Supplementary Table 1 Pneumonitis characteristics.

| **Characteristics** | **N (%) (n = 58)** |
| --- | --- |
| Interval between TRT and TRP, months (range) | 3.58(0.47-11.77) |
| Type of pneumonitis |  |
| RP | 40(69.0) |
| CIP | 6(10.3) |
| Mixed pneumonitis | 12(20.7) |
| CTCAE grade, n (%) |  |
| Grade 1 | 26(44.8) |
| Grade 2 | 32(55.2) |
| Grade 3 | 0(0) |
| Grade 4 | 0(0) |
| Grade 5 | 0(0) |
| Clinical symptom |  |
| Cough | 19(32.8) |
| Sputum | 9(15.5) |
| High temperature | 9(15.5) |
| Chest pain | 2(3.4) |
| Asymptomatic | 27(46.6) |
| Radiotherapy area | 4(6.9) |
| primary focus of the lungs | 4(6.9) |
| mediastinal lymph nodes | 6(10.3) |
| both | 48(82.8) |
| PTV, cm3 (range) | 186.3(41.6-559.7) |
| Median radiotherapy dose,Gy (range) | 54(44-66) |
| Median Radiotherapy fractionation,T (range) | 18(8-30) |
| MLD, Gy (range) | 961.65(43.2-1356.9) |
| Median V5, % | 36.12 |
| Median V10, % | 26.3 |
| Median V20, % | 17.42 |
| Median V30, % | 10.52 |

# TRT: Thoracic Radiotherapy, TRP: Treatment Related Pneumonitis, RP: Radiation Pneumonitis, CIP: checkpoint inhibitor-associated pneumonitis, CTCAE: Common Terminology Criteria for Adverse Events, PTV: Planning Target Volume, MLD: Mean Lung Dose. V5: percent volume of lung receiving ≥5Gy, V10: percent volume of lung receiving ≥10Gy, V20: percent volume of lung receiving ≥20Gy, V30: percent volume of lung receiving ≥30Gy.

# Supplementary Table 2 Characteristics of patients in the two groups and with the χ2 test for categorical variables.

| Variable | Group with pneumonitis (n = 58) | Group with no pneumonitis (n = 28) | *P* value |
| --- | --- | --- | --- |
| Age |  |  | 0.278 |
| ≥60 | 39(67.2) | 22(78.6) |  |
| <60 | 19(32.8) | 6(21.4) |  |
| Gender |  |  | 0.130 |
| male | 47(81.0) | 26(92.9) |  |
| female | 11(19.0) | 2(7.1) |  |
| Smoking |  |  | 0.149 |
| current or former | 32(55.2) | 20(71.4) |  |
| never | 26(44.8) | 8(28.6) |  |
| History of chest operation |  |  | 0.290 |
| no | 49(84.5) | 21(75.0) |  |
| yes | 9(15.5) | 7(25.0) |  |
| KPS |  |  | 0.775 |
| ≥90 | 51(87.9) | 24(85.7) |  |
| <90 | 7(12.1) | 4(14.3) |  |
| Histology |  |  | 0.557 |
| Adenocarcinoma | 23(39.7) | 8(28.6) |  |
| Squamous cell lung carcinoma | 31(53.4) | 17(60.7) |  |
| NSCLC-NOS | 4(6.9) | 3(10.7) |  |
| T stage |  |  | 0.452 |
| T1-2 | 22(37.9) | 13(46.4) |  |
| T3-4 | 36(62.1) | 15(53.6) |  |
| N stage |  |  | 0.958 |
| N0-1 | 6(10.3) | 3(10.7) |  |
| N2-3 | 52(89.7) | 25(89.3) |  |
| M stage |  |  | 0.217 |
| M0 | 38(65.5) | 22(78.6) |  |
| M1 | 20(34.5) | 6(21.4) |  |
| Initial cancer stage |  |  | 0.554 |
| I-II | 3(5.1) | 3(10.7) |  |
| III | 36(62.1) | 18(64.3) |  |
| IV | 19(32.8) | 7(25.0) |  |
| Comorbidities |  |  | 0.112 |
| No | 31(53.4) | 20(71.4) |  |
| Yes | 27(46.6) | 8(28.6) |  |
| Concurrent chemoradiotherapy |  |  | 0.009 |
| No | 36(62.1) | 25(89.3) |  |
| Yes | 22(37.9) | 3(10.7) |  |
| ICIs Timing |  |  | 0.035 |
| Concurrent | 26(44.8) | 6(21.4) |  |
| Sequential | 32(55.2) | 22(78.6) |  |
| Radiotherapy area |  |  | 0.070 |
| primary focus of the lungs | 4(6.9) | 7(25.0) |  |
| mediastinal lymph nodes | 6(10.3) | 3(10.7) |  |
| both | 48(82.8) | 18(64.3) |  |
| PTV, cm3 (range) | 186.30(41.6-559.7) | 205.50(66.9-536.2) | 0.949 |
| Median radiotherapy dose,Gy (range) | 54(44-66) | 60(30-60) | 0.938 |
| Median Radiotherapy fractionation,T (range) | 18(8-30) | 20(6-30) | 0.848 |
| MLD, Gy (range) | 961.65(43.2-1356.9) | 705.30(274.70-1400.9) | 0.026 |
| Median V5, % | 36.12 | 26.35 | 0.092 |
| Median V10, % | 26.30 | 19.09 | 0.076 |
| Median V20, % | 17.42 | 12.83 | 0.037 |
| Median V30, % | 10.52 | 8.04 | 0.117 |

# KPS: karnofsky performance status, ICIs: immune checkpoint inhibitors, NSCLC-NOS: non-small cell lung cancer -not otherwise specified, PTV: Planning Target Volume, MLD: Mean Lung Dose, V5: percent volume of lung receiving ≥5Gy, V10: percent volume of lung receiving ≥10Gy, V20: percent volume of lung receiving ≥20Gy, V30: percent volume of lung receiving ≥30Gy.

# Supplementary Table 3 CT imaging features of treatment-related pneumonitis

| **characteristics** | **N (%) (n = 58)** |
| --- | --- |
| Patch | 58(100) |
| Lung consolidation | 51(87.9) |
| Strip shape | 46(79.3) |
| Ground-glass opacity | 36(62.1) |
| Honeycomb | 24(41.4) |
| Synchronised tumour progression | 7(12.1) |
| Synchronised pleural effusion | 18(31.0) |
| Enlarged non-neoplastic lymph nodes in hilar and mediastinal regions of the lungs | 49(84.5) |
| Thickening of the interlobular septa of the lungs | 22(37.9) |
| Micronodules in the centre of the lung lobules | 4(6.9) |
| Solitary pulmonary nodule or masses | 7(12.1) |
| Pulmonary tree-budding sign | 3(5.2) |
| Thickening of lung bronchial walls | 7(12.1) |
| Deformation of the lung bronchus | 6(10.3) |
| Bronchiectasis | 22(37.9) |
| Reverse pulmonary halo sign (RPH) | 1(1.7) |
| Signs ≥ 6 | 38(65.5) |
| Unilateral lung | 26(44.8) |
| Bilateral lungs | 32(55.2) |
| Involvement of all lobes of the lungs | 1(1.7) |
| Involvement of peripheral subpleural | 54(93.1) |
| Involvement around the mediastinum | 43(74.1) |
| Involvement of peribronchial vessels | 37(63.8) |
| Distribution 1 |  |
| diffuse | 0(0) |
| multifocal | 53(91.4) |
| unifocal | 5(8.6) |
| Distribution 2 |  |
| Keep away from tumours | 3(5.2) |
| peritumour | 6(10.3) |
| all have | 49(84.5) |
| Clear boundaries | 1(1.7) |
| Border ambiguity | 57(98.3) |
| Unilateral lung involvement |  |
| <1/3 | 55(94.8) |
| 1/3-2/3 | 3(5.2) |
| ≥2/3 | 0(0) |
| OP | 58(100) |
| GGO | 37(63.8) |
| NSIP | 8(13.8) |
| AIP/DAD | 58(100) |
| HP | 2(3.4) |
| Bronchitis | 1(1.7) |
| Lung nodule or mass-like | 3(5.2) |
| NOS | 0(0) |

# OP: Organizing Pneumonitis, GGO: Ground Glass Opacity, NSIP: Non-specific interstitial pneumonitis, AIP/DAD: Acute Interstitial Pneumonitis/Diffuse Alveolar Damage, HP: hypersensitivity pneumonitis.

# Supplementary Table 4 Characteristics of patients in the two groups and with the χ2 test for categorical variables.

| Variable | Group with tumour progression (n = 44) | Group with no tumour progression (n = 14) | *P* value |
| --- | --- | --- | --- |
| Age |  |  | 0.362 |
| ≥60 | 31(70.5) | 8(57.1) |  |
| <60 | 13(29.5) | 6(42.9) |  |
| Gender |  |  | 0.599 |
| Male | 35(79.5) | 12(85.7) |  |
| Female | 9(20.5) | 2(14.3) |  |
| Smoking |  |  | 0.865 |
| Current or former | 24(54.5) | 8(57.1) |  |
| Never | 20(45.5) | 6(42.9) |  |
| History of chest operation |  |  | 0.883 |
| No | 37(84.1) | 12(85.7) |  |
| Yes | 7(15.9) | 2(14.3) |  |
| KPS |  |  | 0.495 |
| ≥90 | 38(86.4) | 13(92.9) |  |
| <90 | 6(13.6) | 1(7.1) |  |
| Histology |  |  | 0.532 |
| Adenocarcinoma | 24(54.5) | 6(42.9) |  |
| Squamous cell lung carcinoma | 17(38.6) | 7(50.0) |  |
| NSCLC-NOS | 3(6.8) | 1(7.1) |  |
| Lower lobe of the lungs |  |  | 0.938 |
| No | 35(79.5) | 11(78.6) |  |
| Yes | 9(20.5) | 3(21.4) |  |
| T stage |  |  | 0.663 |
| T1-2 | 16(36.4) | 6(42.9) |  |
| T3-4 | 28(63.6) | 8(57.1) |  |
| N stage |  |  | 0.639 |
| N0-1 | 5(11.4) | 1(7.1) |  |
| N2-3 | 39(88.6) | 13(92.9) |  |
| M stage |  |  | 0.225 |
| M0 | 27(61.4) | 11(78.6) |  |
| M1 | 17(38.6) | 3(21.4) |  |
| Initial cancer stage |  |  | 0.304 |
| I-II | 2(4.5) | 1(7.1) |  |
| III | 26(59.1) | 10(71.4) |  |
| IV | 16(36.4) | 3(21.4) |  |
| Comorbidities |  |  | 0.351 |
| No | 22(50.0) | 9(64.3) |  |
| Yes | 22(50.0) | 5(35.7) |  |
| Concurrent chemoradiotherapy |  |  | 0.407 |
| No | 26(59.1) | 10(71.4) |  |
| Yes | 18(40.9) | 4(28.6) |  |
| ICIs timing |  |  | 0.655 |
| Concurrent | 25(56.8) | 7(50.0) |  |
| Sequential | 19(43.2) | 7(50.0) |  |
| Pneumonitis leads to interruption of treatment |  |  | 0.534 |
| No | 35(79.5) | 10(71.4) |  |
| Yes | 9(20.5) | 4(28.6) |  |
| Interval between TRT and TRP, months (range) | 3.55(0.47-11.77) | 3.67(1.13-9.67) | 0.759 |
| Duration of pneumonitis, months (range) | 3.22(0.43-10.90) | 2.15(0.20-12.63) | 0.424 |
| Lung consolidation |  |  | 0.774 |
| No | 5(11.4) | 2(14.3) |  |
| Yes | 39(88.6) | 12(85.7) |  |
| Strip shape |  |  | 0.484 |
| No | 10(22.7) | 2(14.3) |  |
| Yes | 34(77.3) | 12(85.7) |  |
| Ground-glass opacity |  |  | 0.285 |
| No | 15(34.1) | 7(50.0) |  |
| Yes | 29(65.9) | 7(50.0) |  |
| Honeycomb |  |  | 0.621 |
| No | 25(56.8) | 9(64.3) |  |
| Yes | 19(43.2) | 5(35.7) |  |
| Synchronised tumour progression |  |  | 0.495 |
| No | 38(86.4) | 13(92.9) |  |
| Yes | 6(13.6) | 1(7.1) |  |
| Synchronised pleural effusion |  |  | 0.667 |
| No | 31(70.5) | 9(64.3) |  |
| Yes | 13(29.5) | 5(35.7) |  |
| Enlarged non-neoplastic lymph nodes in hilar and mediastinal regions of the lungs |  |  | 0.883 |
| No | 7(15.9) | 2(14.3) |  |
| Yes | 37(84.1) | 12(85.7) |  |
| Thickening of the interlobular septa of the lungs |  |  | 0.407 |
| No | 26(59.1) | 10(71.4) |  |
| Yes | 18(40.9) | 4(28.6) |  |
| Micronodules in the center of the lung lobules |  |  | 0.967 |
| No | 41(93.2) | 13(92.9) |  |
| Yes | 3(6.8) | 1(7.1) |  |
| Solitary pulmonary nodule or masses |  |  | 0.495 |
| No | 38(86.4) | 13(92.9) |  |
| Yes | 6(13.6) | 1(7.1) |  |
| Pulmonary tree-budding sign |  |  | 0.705 |
| No | 42(95.5) | 13(92.9) |  |
| Yes | 2(4.5) | 1(7.1) |  |
| Thickening of lung bronchial walls |  |  | 0.495 |
| No | 38(86.4) | 13(92.9) |  |
| Yes | 6(13.6) | 1(7.1) |  |
| Distribution of pneumonitis |  |  | 0.088 |
| Unifocal | 39(88.6) | 14(100) |  |
| Multifocal | 5(11.4) | 0(0) |  |
| GGO |  |  | 0.218 |
| No | 14(31.8) | 7(50.0) |  |
| Yes | 30(68.2) | 7(50.0) |  |
| NSIP |  |  | 0.951 |
| No | 38(86.4) | 12(85.7) |  |
| Yes | 6(13.6) | 2(14.3) |  |
| HP |  |  | 0.421 |
| No | 42(95.5) | 14(100) |  |
| Yes | 2(4.5) | 0(0) |  |
| Lung nodule or mass-like |  |  | 0.320 |
| No | 41(93.2) | 14(100) |  |
| Yes | 3(6.8) | 0(0) |  |

# KPS: karnofsky performance status, ICIs: immune checkpoint inhibitors, NSCLC-NOS: non-small cell lung cancer-not otherwise specified, TRT: Thoracic Radiotherapy, TRP: Treatment Related Pneumonitis,GGO: Ground Glass Opacity, NSIP: Non-specific interstitial pneumonitis, HP: hypersensitivity pneumonitis.
